# Rapid and Efficient Colony-PCR for High Throughput Screening of Genetically Transformed *Chlamydomonas reinhardtii*

**DOI:** 10.3390/life10090186

**Published:** 2020-09-10

**Authors:** Serge Basile Nouemssi, Manel Ghribi, Rémy Beauchemin, Fatma Meddeb-Mouelhi, Hugo Germain, Isabel Desgagné-Penix

**Affiliations:** 1 Department of Chemistry, Biochemistry and Physics, Université du Québec à Trois-Rivières, 3351, boul. des Forges, C.P. 500, Trois-Rivières, QC G9A 5H7, Canada; serge.nouemssi@uqtr.ca (S.B.N.); Manel.Ghribi@uqtr.ca (M.G.); Remy.Beauchemin@uqtr.ca (R.B.); Fatma.Meddeb@uqtr.ca (F.M.-M.); Hugo.Germain@uqtr.ca (H.G.); 2 Groupe de Recherche en Biologie Végétale, Université du Québec à Trois-Rivières, 3351, boul. des Forges, C.P. 500, Trois-Rivières, QC G9A 5H7, Canada

**Keywords:** high-throughput screening, microalgae, colony-PCR method, genetic transformation, *Chlamydomonas reinhardtii*, synthetic biology

## Abstract

Microalgae biotechnologies are rapidly developing into new commercial settings. Several high value products already exist on the market, and biotechnological development is focused on genetic engineering of microalgae to open up future economic opportunities for food, fuel and pharmacological production. Colony-polymerase chain reaction (colony-PCR or cPCR) is a critical method for screening genetically transformed microalgae cells. However, the ability to rapidly screen thousands of transformants using the current colony-PCR method, becomes a very laborious and time-consuming process. Herein, the non-homologous transformation of *Chlamydomonas reinhardtii* using the electroporation and glass beads methods generated more than seven thousand transformants. In order to manage this impressive number of clones efficiently, we developed a high-throughput screening (HTS) cPCR method to rapidly maximize the detection and selection of positively transformed clones. For this, we optimized the *Chlamydomonas* transformed cell layout on the culture media to improve genomic DNA extraction and cPCR in 96-well plate. The application of this optimized HTS cPCR method offers a rapid, less expensive and reliable method for the detection and selection of microalgae transformants. Our method, which saves up to 80% of the experimental time, holds promise for evaluating genetically transformed cells and selection for microalgae-based biotechnological applications such as synthetic biology and metabolic engineering.

## 1. Introduction

*Chlamydomonas reinhardtii* is a common single-celled photosynthetic microalga, populating fresh water and moist soil. It is also known for easy isolation, cultivation, and transformation [1,2]. This unicellular, eukaryotic, green microalga strain has a well-characterized genome [3] and it has been commonly used to study a number of biological processes, which makes it a model organism for biotechnological studies. *C. reinhardtii* has been widely used in basic and applied sciences and it is considered an ideal host and cell-factory for the production of high-added value bioproducts [4,5,6]. In addition, because of its rapid growth and high biomass production at low cost, *C*. *reinhardtii* has attracted the interest of several biotechnologists. Nevertheless, the transformation and selection remain the decisive stages in using *C. reinhardtii* microalgae as a biotechnological platform [7]. Several genetic transformation methods have been established to introduce foreign DNA fragments into the *C. reinhardtii* including electroporation, glass beads, silicon carbide whiskers, microparticle bombardment and Agrobacterium-mediated transformation [8,9,10,11]. However, the integration of exogenous DNA into the nuclear genome predominantly occurs randomly via Non-Homologous-End-Joining (NHEJ), leading to a large and heterogeneous population of transformed cells (tens of thousands) with varied expression levels. Moreover, the integrated foreign genes or cassettes may be subjected to deletions, genomic rearrangements and insertions of short DNA fragments [12,13], in addition to the epigenetic silencing phenomenon that decrease or suppress the expression of the transgenes. Such events require a meticulous high throughput screening (HTS) technique to handle the high number of the genetically transformed cells with desirable expression level [8,14,15,16,17].

The traditional method of initial screening of microalgae transformants is based on selectable markers such as antibiotic resistance and fluorescence, that are commonly used or a combination of bicistronic mRNA constructs that allows expression of a gene of interest (GOI) and a selectable marker [7,18,19,20,21,22,23,24,25,26,27,28,29,30]. However, the screening method based on antibiotic resistance approach remains limited due to its large number of false positives. On other hand, fluorescence reporter screening has been used in several studies and has demonstrated a high potential in order to select positively transformed microalgae based on their fluorescence emission [21,26,27,28,29]. This method seems, however, inconvenient for laboratories indisposed of the fluorescent agar plate reader. Additionally, strains with low fluorescence could not be easily detected. An additional screening method, named colony polymerase chain reaction (cPCR) is used to select antibiotic-resistant transformants that harbor copies of the integrated expression cassettes [31]. Although DNA purification is still used, the Chelex method seems to be way easier and faster. cPCR using Chelex for DNA extraction is a powerful technique that bypasses DNA purification [31]. This method relies on the selectivity of PCR amplification to determine whether a microorganism of interest integrates the target inserted DNA. For bacteria and yeast, simply adding a small portion of cell colony directly to a PCR master mix will introduce enough template DNA for amplification [32]. cPCR is challenging with microalgae because of their complex cell wall structure that precludes efficient extraction of their genetic material leading to a more laborious and time-consuming PCR, especially when the number of transformants to be screened is large. Another challenging issue is to maintain thousands of clones by subculturing and managing them using single Eppendorf and PCR tubes for the processing of transformants by cPCR. Hence, the need to develop an optimized high throughput screening (HTS) cPCR method, to maximize the detection and selection of hundreds or even thousands of positive transformed clones rapidly, becomes an urgent requirement. Therefore, the efficiency is critical: every improvement in workflow efficiency leads to significant savings in time. Eliminating pipetting steps reduces the potential for contamination and error, and minimizes repetitive strain-induced injury. Simplifying the screening procedure also enables better standardization between different laboratory personnel, which can be crucial for screening a huge number of transformants.

Here, we report a rapid and an optimized method for handling a high number of *C. reinhardtii* transformants with embedded foreign genes or cassettes in the genomic DNA. We describe details (steps and time savings) of this rapid and specific molecular technique and high-throughput (HT) PCR samples loading onto agarose gel for electrophoresis and the analysis of a high number of genetically transformed microalgae. The effectiveness of the proposed procedure, 96-well plate PCR (named here, HT), was compared with single tube PCR (named here, standard (ST)) screening.

## 2. Materials and Methods

### 2.1. C. reinhardtii Strain, Plasmid and Primers Design

**Figure A1:**
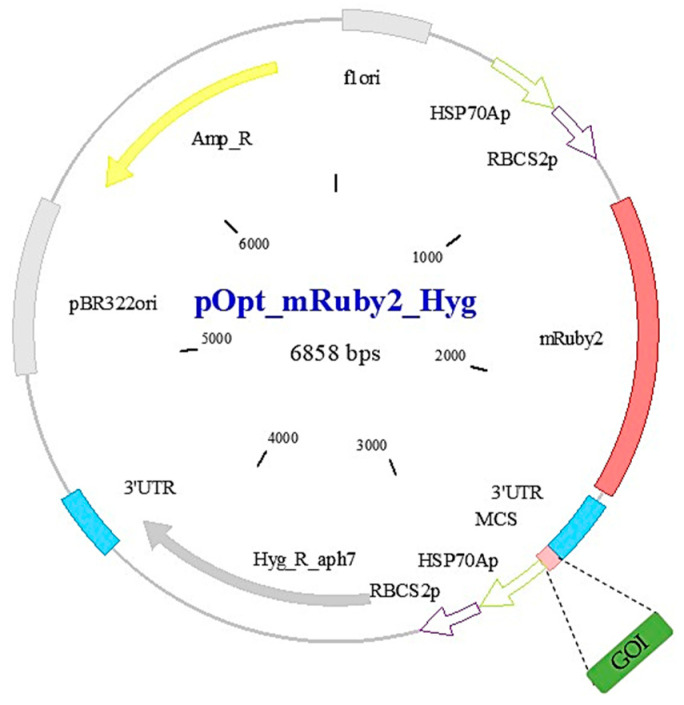
Vector map of the recombinant pOpt_mRuby2_Hyg vector [21] containing gene of interest (GOI). The vector map was made using Clone Manager suite 7 software.

*C. reinhardtii* strain C-137 wild type (wt) and the pOptimized_mRuby2_Hygromycin (pOpt_mRuby2_Hyg) [21] cloning vector were purchased from the Chlamydomonas Resource Center (Minneapolis, MN, USA) [33]. Recombinant plasmid construct, pOpt_mRuby2_Hyg containing gene of interest (GOI) (Appendix A Figure A1) was made using Gibson assembly strategy. Transformed *E. coli* DH5 alpha cells, bearing the pOpt_mRuby2_Hyg-GOI expression, were selected on LB-agar with 100 µg/mL Ampicillin. Plasmid DNA was isolated using a Biobasic plasmid extraction kit (Cat no# BS614). Plasmid DNA was then used to ascertain the integrity of the nucleotide sequence by next gene sequencing (at MGH CCIB DNA CORE). The nuclear phosphoglycerate kinase (PGK), a housekeeping gene [34] (GeneBank ID: AF244144.1), is used as a positive PCR control. The oligonucleotide primers used in this study were synthesized by Integrated DNA Technologies (San Diego, CA, USA) [35]. The following primers were used to amplify nuclear (Nuc) PGK and GOI genes to obtain PCR product of 944 and 506 bp, respectively:

Nuc_PGK_F: 5′AGTCCGAACAACCCACTTAC 3′;
Nuc_PGK_R: 5′ CAGAGCTCAGGAGGTGAAATAG 3′;
GOI_F: 5′ GCTGGGCATCAAGAAGACCG 3′;
GOI_R: 5′ CCTGCGTGTAGTTGTTCGGG 3.’

### 2.2. C. reinhardtii Growth and Transformation Conditions

Unless otherwise stated, cells were cultivated mixotrophically at 25 °C in tris-acetate phosphate (TAP) medium under moderate and continuous white fluorescent light at the intensity of 50 µmol photons m^−2^ s^−1^ in shake flasks or on agar plates with a relative humidity (Rh) of 50%.

Herein, electroporation method was performed for transformation as described in [10,36,37] with slight modification. *C. reinhardtii* cells were transformed using the Bio-Rad Genepulser XcellTM electroporation machine and 4 mm cuvette under the following parameters: voltage 0.5 kV; capacitance 50 µF; resistance 800 Ω.

Briefly, liquid state cells were grown in 30 mL TAP culture medium in a 125 mL Erlenmeyer flask with an initial Optical Density at 750 nm (OD_750nm_) of 0.1 (1 × 10^5^ cells/mL) with gentle shaking (100 rpm) to a final OD_750nm_ of 0.7 (7 × 10^6^ cells/mL). Cells were harvested by centrifugation at 7000× *g* for 5 min and then washed three times by resuspending the pellet in 5 mL of Max Efficiency™ Transformation Reagent for Algae (Invitrogen Cat no# A24229) and centrifuged in the same conditions as in the harvesting step. The samples were incubated on ice for 10 min prior to electroporation which was performed by applying an electric pulse using 250 μL of *C. reinhardtii* cells and 500 ng of linearized purified plasmid. Transgenic strains were resuspended in 5 mL of TAP liquid medium supplemented with 40 mM sucrose (TAP/sucrose) and then incubated at 25 °C with gentle shaking (100 rpm) for 22 h under continuous light. After incubation, the transformed cells were harvested by centrifugation at 7000× *g* for 5 min and resuspended in 250 μL of Max Efficiency. Then, spread on TAP agar media supplemented with Hygromycin (10 µg/mL) and incubated in a growth chamber for around 5 to 7 days.

When single clones appeared on agar Petri dish, total number of transformants on each plate was counted using OpenCFU software [38] to determine transformation efficiency.

### 2.3. Strains Layout and Selection Method

Hygromycin-resistant isolated transformants were selected randomly and colonies were sub-cultured on TAP agar plate media supplemented with Hygromycin (Hyg) freshly prepared to verify and ensure their growth stability.

**Figure 1:**
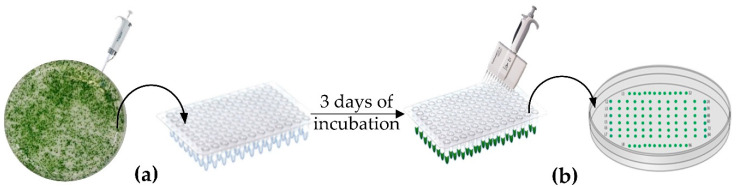
First round of selection and maintenance of transformants. (**a**) Selection of isolated transformants from tris-acetate phosphate (TAP) agar plate using 10 µL tips after 7 days of incubation and inoculation in liquid media in 96-well plate, (**b**) Sub-culturing and maintaining of selected clones according to the model template of the 96-well plate.

In order to achieve a high throughput screening by cPCR, we have optimized the layout of the clones on the culture media according to the model of a 96-well plate which constitutes the template used for this work as shown in Figure 1. The current approach allows to randomly select the maximum number of clones and to easily maintain them by successive sub-culturing on TAP media supplemented with Hygromycin (hyg).

The first selection step was done by picking-up the isolated clones from the transformed *C. reinhardtii* cells using sterile micropipette tips, and resuspending them in 200 μL of TAP containing 10 µg/mL of Hyg liquid medium initially distributed in a cover sterile 96-well plate (Thermofisher Cat no# 655180) (Figure 1a). Cells in suspension were incubated in the growth chamber under gentle shaking for 3 days. After incubation, 2 μL of the suspended cells were withdrawn using a 12-multi-channel micropipette and added, in the same schema as in the 96-well plate, to TAP agar media containing 10 µg/mL of Hyg prepared in a large Petri dish (150 mm × 15 mm) (Fisher Scientific Cat no# 249964) (Figure 1b) and incubated for 7 days at 25 °C under same conditions described in Section 2.2. The selected clones were maintained by several round of subculturing using the same approach as described previously with a 12-multi-channels micropipette every 3 to 4 weeks. Multiple rounds of screening with antibiotics helped to eliminate false positive and verify the stability of the transgene over time.

### 2.4. High Throughput Genomic DNA Extraction

**Figure 2:**
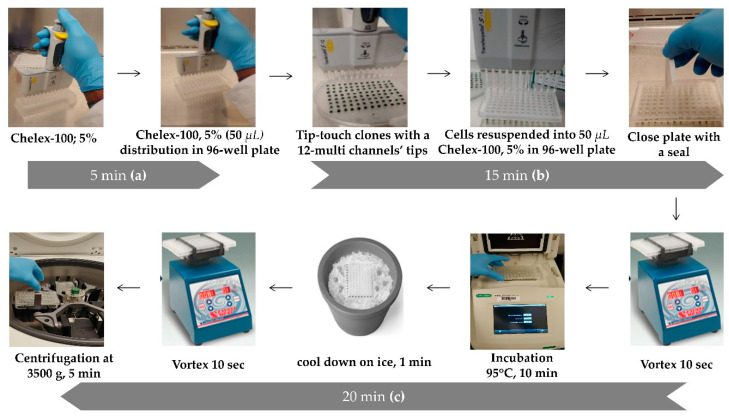
High throughput DNA extraction and time correlation for each step. The protocol is represented in 3 steps corresponding to the first 5 min (**a**), followed by the next 15 min (**b**) and onto the last 20 min (**c**).

Genomic DNA extraction was done according to the protocol described by Cao et al. [31] with slight modifications. For a better handling of the high number of clones obtained after the transformation and subculture of hygromycin-resistant colonies, we developed a high-throughput DNA extraction procedure. For this, we used the big Petri dishes (150 mm × 15 mm) (Fisher Scientific Cat no# 249964, Waltham, MA, USA) containing 96 recombinant colonies, laid out for 96-well plate (as shown in Figure 1) and for a 12 multi-channels micropipette (P200). This technique helps to save time by extracting genomic DNA from 96 different clones at the same time (counting the untransformed wild-type as a negative control) (Figure 2).

First, 5 mL of 5% Chelex-100 solution was prepared as described by [31] and a volume of 50 µL was dispensed, as shown in Figure 2a, in each well of PCR 96 well plate using 12-multi-channels micropipette (or dispensing pipette). Then, with the extremity of the 12-multi-channels micropipette’s tips, cells were transferred by tip-touching the clones (around 1 mm in diameter) from the initial agar plate, and resuspended in the first row of the 96-well plate containing 50 µL of 5% Chelex-100 as described in Figure 2b. This step is repeated for each row (i.e., 8 times from line A to line H of the 96-wells plate) following the order on the agar plate and then close the 96-wells plate with an appropriate seal (Thermofisher Scientific Cat no# AB-0558). The mixture was vigourously vortexed for 10 s and incubated at 95 °C (in the PCR thermocycler) for 10 min (Figure 2c). After incubation, the plate was cool down on ice for 1 min and vortexed again for 10 s. Finally, the 96-well plate containing the extracted DNA was centrifugated at 3500× *g* for 5 min at room temperature using a 96-wells plate centrifuge as described in Figure 2c. A volume of 2 µL of supernatant of each well was used as DNA samples for PCR reaction (final volume of 25 µL). The concentration of the extracted genomic DNA of the different *C. reinhardtii* transformants were measured using a plate reader (Biotek synergy^H1^ microplate reader) at OD_260nm_ (Appendix A Table A1) and DNA samples were stored at −20 °C for further use.

### 2.5. HT PCR and HT Sample Loading onto Adapted Agarose Gel

**Figure 3:**
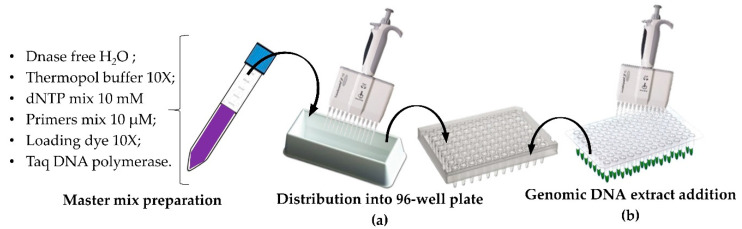
PCR reaction mix preparation and aliquoting. (**a**) master mix reaction preparation and distribution into 96-well plate; (**b**) DNA sample addition in the PCR reaction mix.

The PCR reaction preparation has been performed using DNA Taq polymerase (New England Biolabs, Whitby, ON, Canada, cat no# M0273) with the standard thermopol buffer. Reaction mix (25 µL/PCR), total volume required to assay 96 clones in the same PCR cycle (2.4 mL), was prepared according to NEB builder’s protocol with slight modification (Appendix A Table A2). Once prepared, the mix was poured into a sterile reservoir and a volume of 23 µL of reaction mix was aliquoted using a 12-multi-channels micropipette into a 96-well PCR plate (Figure 3a) and 2 µL of DNA sample of different *C. reinhardtii* transformants were added into each well (Figure 3b). The plate was sealed (Thermofisher Scientific Cat no# AB-0558) to avoid evaporation and then placed into a thermocycler with a specific running cycle to amplify the GOI. The reaction conditions were 95 °C for 5 min, followed by 34 cycles of denaturation at 95 °C for 30 s, annealing at 54 °C for 30 s and extension 68 °C for 1 min. The last step of the reaction was a final elongation at 68 °C for 5 min and stored at 4 °C.

In order to be able to easily load all the amplification products on an agarose gel, two large electrophoretic tanks containing an adapted 52 wells comb were used. The loading dye buffer 10X was prepared with bromophenol blue and glycerol as described by Le Gouill and al., [39] with slight modifications. It was initially added directly into the PCR reaction mix (as shown in Figure 3) before the amplification step so that the PCR amplified samples were loaded directly onto the 1% agarose gel using a 12-multi-channels micropipette.

This experimental step allowed us either to highlight and confirm the HTS genomic DNA (gDNA) extraction protocol by amplifying a 944 bp fragment of the genomic DNA of Chlamydomonas that is specific to the PGK gene (as reference gene and positive control of the genomic DNA extraction), or to select the positive clones during the screening experiment by HTS-cPCR. Both primer pairs (for PGK and GOI) were used in the same PCR reaction. Positive transformants were identified by amplifying a 506 bp fragment of the gene of interest GOI. This method allows to screen rapidly and select a high number of genetically transformed *C. reinhardtii* microalgae.

## 3. Results

### 3.1. Strains Layout and HTS DNA Extraction

**Figure 4:**
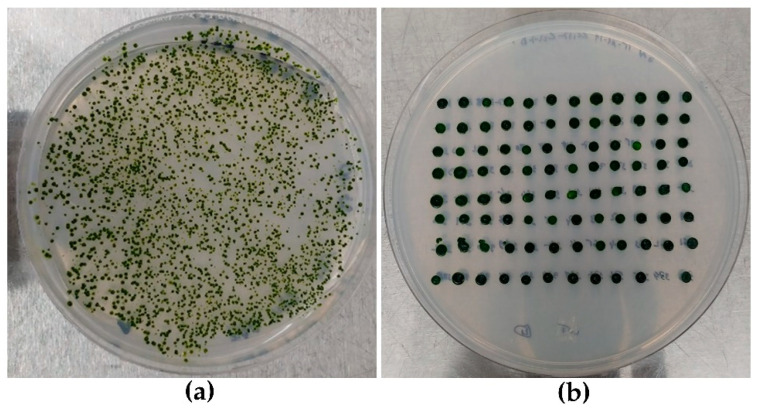
Antibiotic selective TAP agar plate containing genetically transformed *C. reinhardtii*. (**a**) Colonies obtained after 7 days incubation of genetically transformed cells; (**b**) transformants layout as 96-well plate on culture medium.

A high transformation efficiency of up to 1 × 10^6^ CFU/μg of linear plasmid DNA was obtained using the electroporation method. After 5 to 7 days of incubation on TAP agar plate supplemented with 10 µg/mL of hygromycin, we obtained more than 7000 *C. reinhardtii* transformants (Figure 4a). A total of 1440 transformants were selected randomly and cultured according to the layout on TAP agar following a 96-well plate model (Figure 4b). The use of the 96-well format template is modern state of art for different Chlamydomonas laboratories and it facilitates a better management of high numbers of clones. This layout of transformants on the TAP agar media allows for the rapid round culturing of the clones and for the easy pick-up of colonies for the high throughput genomic DNA extraction.

After the high throughput extraction, the genomic DNA concentration was measured using the Take three plate into the plate reader (Biotek synergy|^H1^ microplate reader) (Appendix A Table A1). This assay was performed to ensure that the DNA concentration obtained following our high throughput DNA extraction was relatively uniformed across all colony tested and within each cPCR reaction and that the DNA quality was good [40].

The range of DNA concentration of the transformants was between 21 to 317 ng/µL and showed high purity according to the OD ratio 260/280 nm (Appendix A Table A1) [41]. Genomic DNA extraction can also be performed from liquid cell cultures from the 96-well plate after 3 days incubation in selective media (Figure 1a) for cPCR amplification (data not shown).

### 3.2. High-Troughput Screening cPCR and Revelation

**Figure 5:**
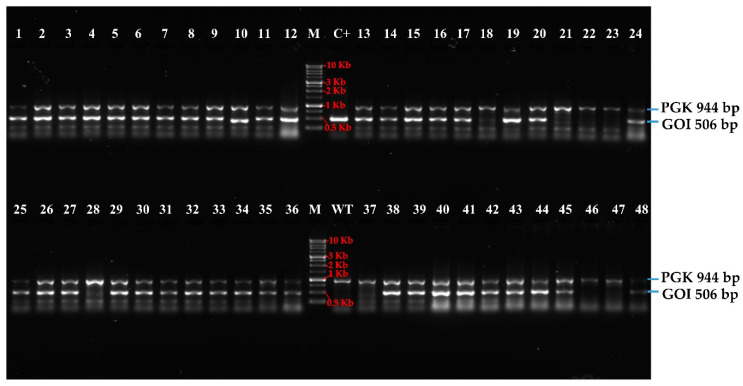
Example of stained SYBR safe agarose gel (1%) showed amplicon of 48 samples: (**PGK**) 48 nuclear phosphoglycerate kinase (PGK) gene PCR amplification at 944 bp; (**GOI**) 48 gene of interest (GOI) PCR amplification at 506 bp. PCR samples were migrated at 100 volt/cm for 60 min. Colony numbers are shown on top of the wells M: 1kb DNA ladder, Froggabio Cat. DM010-R500; wt: wild type (untransformed *C. reinhardtii*) extracted using the same standard protocol as the transformed clones; C+: positive control is plasmid construct (pOpt_mRuby_Hyg-GOI).

Genomic DNA samples from 1440 transgenic *C. reinhardtii* and wild type were extracted using HTS extraction method as described in the materials and methods section. The nuclear PGK gene was used as reference to check the quality, reliability, and specificity of extracted genomic DNA. A fragment of 944 bp of the PGK gene was amplified to provide an example from 48 *C. reinhardtii* transformed and untransformed (named here wt) clones (Figure 5). The result showed the efficiency of HT genomic DNA extraction and HT amplification method. Forty-eight positive amplicons of PGK gene were obtained out of 48 cPCR. Thus, the specific high throughput screening and selection of mutants positively transformed becomes easier using the specific primers in the same reactional mix for the amplification of the gene of interest in the same genomic DNA samples. In addition, DNA extracted sample can be used for PCR amplification after storage at −20 °C.

In the same order, the amplification of a fragment of 506 bp related to the GOI from 48 transformed clones showed 40 positive clones (Figure 5). The number of PCR-positive transformants/number of transformants analyzed ratio is 646/1336. Thus, the percentage of positive colonies is 48.3% (Table 1).

By using this HTS-screening method, we were able to obtain 646 positive transformants over 1336 viable transformants selected after transformation (Table 1).

## 4. Discussion

According to Cao et al., [31], colony-PCR (or cPCR) is known as a highly efficient method of genetic detection. This standard gold technique also has diverse advantages for the screening and selection of genetically transformed cells. Regarding microalgae transformation, this method is shown to be efficient and more specific for the screening and selection of positive transgenes in several genetically transformed green algae such as *C. reinhardtii, Chlorella pyrenoidosa* to name a few [42,43,44,45,46].

The transformation of *C. reinhardtii* using the electroporation method led to a high number of transformants with a transformation efficiency of 1 × 10^6^ CFU/μg of linear plasmid DNA [37]. Because of the random integration of a transgene into the *C. reinhardtii* nuclear genome, the fact that transformants can grow on TAP agar supplemented with hygromycin does not necessarily imply that the clones also integrated the gene of interest. Thus, it is very important to screen a high number of clones to increase the chances of selecting transformants which could have integrated one or multiple copies of the exogenous gene of interest and feature high transgene expression [14].

In order to tackle this issue, we optimized a high throughput screening (HTS) method based on our standard cPCR protocol. In this study, we showed that this HTS screening/selection method is easier, more practical and efficient to test and manage a high number of transformants either for cPCR assay, loading samples (onto agarose gel) or culture maintenance of transformants. Our developed method was compared to the standard screening (STS) method based on individual selection and maintenance, DNA extraction and PCR of transformants clone by clone. This HTS method is not only fast and efficient, but also less expensive and requires less working-time, unlike the STS method, which is more laborious. In Table 2, we present the working-time comparison determined between the STS method and HTS method on 96 clones by cPCR during this study. This comparison was done mainly for the principal steps of the experiment.

The standard cPCR method is clearly a highly time-consuming step when the number of clones tested is elevated to hundreds (for example 96 clones). The HTS-PCR is a six-fold time-saving method which makes work less laborious and cheaper in terms of time, material or money. Moreover, using this method allowed us to avoid mistakes that can occur while working with single tube, and increase the reliability of the result as well as positive clones selected. Indeed, the percentage of positive colonies obtained using HTS was 48.3%, compared to the 20% using conventional screening. Besides all the advantages given by this method, it is important to note that the HTS-PCR is reflected positively in the ecological plan due to reduction of consumable and material used that could be a threat for the environment (such as single Eppendorf tubes and single PCR tubes to 96 wells plates i.e., less plastics). Finally, the layout template and the use of multi-channels pipets for culturing and maintaining the positive clones could be a useful technique for any genetic screening tool such as Southern blotting.

## 5. Conclusions

In this work, we described the high throughput screening of *C. reinhardtii* genetically transformed cells and established a promising, reliable and rapid routine colony-PCR (cPCR) technique. This could help for easier and enhanced screening and selection of massive number of positive transformed cells, which could serve for academic and industrial uses. This technique also allows the manipulation of a high number of positively transformed cells accurately. In addition, more errors can be introduced from the standard technique which becomes very laborious and requires a lot of working-time when the number of clones to be tested becomes large.

In further studies, we aim to implement the HTS detection system for gene expression, based on the use of fluorescent proteins. We hope that this fast model HTS system could facilitate the first step of selecting the supposedly positive clones directly on agar plate before proceeding by any further analysis.

## Figures and Tables

**Table 1:** Number of transgenes selected by the high-throughput screening (HTS)-screening method.

Number of Colonies Counted After Electroporation	Number of Clones Selected Randomly	Viable Clones After Six Round Sub-Culturing (% of Viable Cells)	Positive Clones After HTS-Colony-PCR (% of Positive Colonies)
7000	1440	1336 (93)	646 (48, 3)

**Table 2:** Working-time comparison between standard screening (STS; 96 colonies) and high throughput screening (HTS; 96 colonies) cPCR method.

Steps	STS	HTS	Difference
	Time (min)		
DNA extraction	360	40	320
Mix PCR preparation and distribution	150	35	115
Gel preparation and sample loading	150	20	130
**Total**	660	95	565

**Figure A2:**
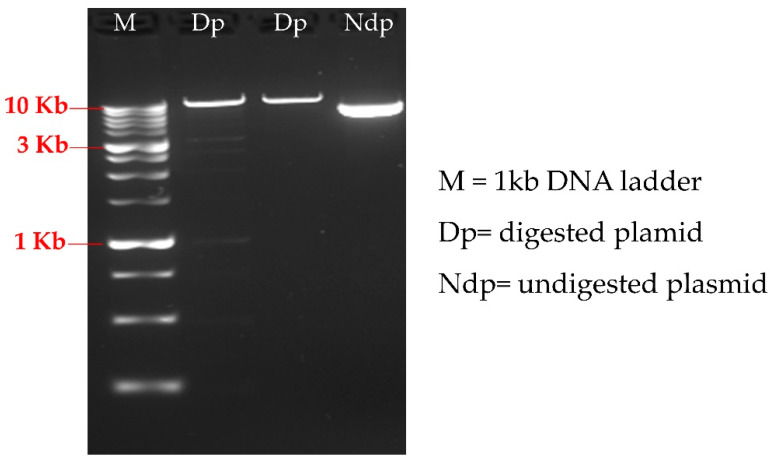
Linearization of the plasmid pOpt_mRuby2_Hyg containing gene of interest using the restriction enzyme ScaI. The 1kb DNA ladder used is from FroggaBio (Concord, ON, Canada) Cat. DM010-R500

## Appendix A

Our study used the following vector (Figure A1).

The results for the DNA concentration are displayed in Table A1.

**Table A1:** Genomic DNA concentration obtained after HT DNA extraction in 96-well plate. Here we show 48 DNA extracted samples.

Sample Read#	Well ID	Location	260 Raw	280 Raw	320 Raw	260	280	260/280	ng/µL
1	SPL1	A2	0.167	0.134	0.074	0.09	0.057	1.562	89.714
2	SPL2	A3	0.167	0.124	0.056	0.107	0.065	1.639	106.894
3	SPL3	B2	0.158	0.131	0.084	0.07	0.044	1.569	69.743
4	SPL4	B3	0.19	0.143	0.06	0.126	0.08	1.569	126.219
5	SPL5	C2	0.129	0.101	0.057	0.067	0.041	1.633	67.486
6	SPL6	C3	0.17	0.129	0.063	0.102	0.063	1.629	102.019
7	SPL7	D2	0.121	0.092	0.05	0.067	0.04	1.675	66.743
8	SPL8	D3	0.117	0.094	0.059	0.054	0.033	1.653	53.816
9	SPL9	E2	0.112	0.088	0.051	0.056	0.034	1.647	56.21
10	SPL10	E3	0.141	0.105	0.051	0.087	0.052	1.684	86.768
11	SPL11	F2	0.101	0.081	0.049	0.048	0.029	1.622	47.791
12	SPL12	F3	0.114	0.092	0.049	0.061	0.04	1.526	60.933
**B**	**SPL13**	**G2**	**0.042**	**0.043**	**0.036**	**0**	**0**	**0**	**0**
13	SPL1	A2	0.156	0.117	0.053	0.1	0.061	1.631	99.51
14	SPL2	A3	0.09	0.074	0.051	0.034	0.02	1.758	34.371
15	SPL3	B2	0.24	0.173	0.06	0.175	0.11	1.598	175.279
16	SPL4	B3	0.117	0.092	0.054	0.059	0.035	1.682	58.657
17	SPL5	C2	0.114	0.091	0.055	0.055	0.033	1.651	55.252
18	SPL6	C3	0.12	0.092	0.05	0.066	0.04	1.667	65.907
19	SPL7	D2	0.393	0.279	0.07	0.317	0.205	1.549	317.437
20	SPL8	D3	0.103	0.082	0.049	0.05	0.03	1.63	49.642
21	SPL9	E2	0.1	0.081	0.051	0.044	0.027	1.66	44.324
22	SPL10	E3	0.104	0.082	0.047	0.053	0.032	1.677	53.017
23	SPL11	F2	0.145	0.111	0.057	0.083	0.051	1.624	82.756
24	SPL12	F3	0.164	0.143	0.106	0.054	0.034	1.604	54.168
**B**	**SPL13**	**G2**	**0.045**	**0.042**	**0.039**	**0**	**0**	**0**	**0**
25	SPL1	A2	0.118	0.093	0.049	0.066	0.041	1.593	65.524
26	SPL2	A3	0.122	0.094	0.051	0.067	0.04	1.666	67.201
27	SPL3	B2	0.174	0.126	0.05	0.12	0.074	1.63	119.823
28	SPL4	B3	0.259	0.191	0.068	0.186	0.119	1.562	186.418
29	SPL5	C2	0.233	0.169	0.054	0.174	0.111	1.566	174.01
30	SPL6	C3	0.132	0.102	0.051	0.077	0.048	1.587	76.95
31	SPL7	D2	0.071	0.061	0.046	0.022	0.013	1.751	21.983
32	SPL8	D3	0.114	0.09	0.055	0.055	0.033	1.672	54.91
33	SPL9	E2	0.072	0.061	0.044	0.024	0.014	1.723	24.317
34	SPL10	E3	0.083	0.067	0.044	0.034	0.02	1.689	34.399
35	SPL11	F2	0.145	0.114	0.065	0.076	0.046	1.637	75.822
36	SPL12	F3	0.105	0.084	0.049	0.053	0.033	1.618	52.875
**B**	**SPL13**	**G2**	**0.042**	**0.041**	**0.038**	**0**	**0**	**0**	**0**
37	SPL1	A2	0.116	0.093	0.05	0.063	0.041	1.547	63.025
38	SPL2	A3	0.277	0.201	0.062	0.21	0.136	1.545	209.66
39	SPL3	B2	0.15	0.114	0.051	0.095	0.06	1.577	95.231
40	SPL4	B3	0.354	0.259	0.064	0.286	0.192	1.49	285.522
41	SPL5	C2	0.216	0.163	0.061	0.151	0.098	1.532	150.736
42	SPL6	C3	0.113	0.088	0.045	0.064	0.04	1.602	64.316
43	SPL7	D2	0.206	0.155	0.057	0.145	0.096	1.517	144.948
44	SPL8	D3	0.327	0.241	0.074	0.248	0.164	1.514	247.814
45	SPL9	E2	0.177	0.135	0.058	0.114	0.074	1.547	113.956
46	SPL10	E3	0.154	0.118	0.051	0.099	0.065	1.53	98.815
47	SPL11	F2	0.381	0.282	0.097	0.277	0.18	1.538	276.991
48	SPL12	F3	0.157	0.118	0.052	0.101	0.063	1.609	100.925

B = The bold raw represent blank negative control (distilled water).

Our study used the following master mix preparation (Table A2).

**Table A2:** Reaction master mix preparation (source NEB builder [47]).

Component	25 μL Reaction	Final Concentration
10× ThermoPol Reaction Buffer	2.5 µL	1X
10 mM dNTPs	0.5 µL	200 µM
10 µM Forward Primer mix (PGK/GOI)	0.5 µL	0.2 µM (0.05–1 µM)
10 µM Reverse Primer mix (PGK/GOI)	0.5 µL	0.2 µM (0.05–1 µM)
10× Loading Dye Buffer	1 µL	0.4X
Template DNA	2 µL	<1000 ng
*Taq* DNA Polymerase	0.125 µL	1.25 units/50 µL PCR
Nuclease-free water	To 25 µL	
